# Using Bayesian networks to guide the assessment of new evidence in an appeal case

**DOI:** 10.1186/s40163-016-0057-6

**Published:** 2016-05-25

**Authors:** Nadine M. Smit, David A. Lagnado, Ruth M. Morgan, Norman E. Fenton

**Affiliations:** 1 Department of Security and Crime Science, University College London, 35 Tavistock Square, London, WC1H 9EZ UK; 2 Centre for the Forensic Sciences, University College London, 35 Tavistock Square, London, WC1H 9EZ UK; 3 Department of Experimental Psychology, University College London, 26 Bedford Way, London, WC1H 0AP UK; 4 School of Electronic Engineering and Computer Science, Queen Mary University of London, Mile End Road, London, E1 4NS UK

**Keywords:** Bayesian networks, Bayesian inference, Audio evidence, Forensic reasoning, Appeal

## Abstract

When new forensic evidence becomes available after a conviction there is no systematic framework to help lawyers to determine whether it raises sufficient questions about the verdict in order to launch an appeal. This paper presents such a framework driven by a recent case, in which a defendant was convicted primarily on the basis of audio evidence, but where subsequent analysis of the evidence revealed additional sounds that were not considered during the trial. The framework is intended to overcome the gap between what is generally known from scientific analyses and what is hypothesized in a legal setting. It is based on Bayesian networks (BNs) which have the potential to be a structured and understandable way to evaluate the evidence in a specific case context. However, BN methods suffered a setback with regards to the use in court due to the confusing way they have been used in some legal cases in the past. To address this concern, we show the extent to which the reasoning and decisions within the particular case can be made explicit and transparent. The BN approach enables us to clearly define the relevant propositions and evidence, and uses sensitivity analysis to assess the impact of the evidence under different assumptions. The results show that such a framework is suitable to identify information that is currently missing, yet clearly crucial for a valid and complete reasoning process. Furthermore, a method is provided whereby BNs can serve as a guide to not only reason with incomplete evidence in forensic cases, but also identify very specific research questions that should be addressed to extend the evidence base and solve similar issues in the future.

## Background

Extensive research is being undertaken in various scientific disciplines to extend the evidence base that can help the detection, collection, and analysis of forensic evidence (INTERPOL 2013). Such empirically based knowledge, together with experiential insights, can begin to address questions regarding the way in which evidence behaves [e.g. (French and Morgan 2015)] to assess the probability of observations under proposed defence and prosecution hypotheses. In practice, the personal opinion of the expert, based on their experience, is generally accepted by the court as valid evidence (Morgan and Bull 2007; R v. Weller 2010; The Law Commission 2011). Even though this kind of expert judgment can be informative, especially in the absence of an empirical evidence base, these subjective arguments raise questions about issues such as the validation of the arguments put forward by the expert (National Research Council 2009) and susceptibility to cognitive influences on the interpretation of evidence under uncertainty (Kassin et al. 2013; Nakhaeizadeh et al. 2014). Similarly, interpretation issues can be faced by the court when experts do provide empirical evidence, due to the lack of agreement and consistency in the application of current interpretation approaches (National Research Council 2009), and the difficulties in presenting the reasoning processes of the scientists transparently in court (Sjerps and Berger 2012).

### Approaches to interpretation

To offer a scientific methodological approach to the forensic interpretation and decision-making process, the *case assessment and interpretation* (*CAI*) model was developed in the late nineties by Cook et al. (1998b). This framework requires one to make a structured and systematic assessment of the requirements, questions, and outcomes of the analysis to optimise the contribution of the evidence to the case (Jackson et al. 2013). Because the formulation of the hypotheses is of high significance in the interpretation of the evidence (Fenton et al. 2014), it is important to consider the hypotheses that the different stakeholders realistically can address, using their expertise, to come to a defendable conclusion. Therefore, Cook et al. developed the so-called *hierarchy of propositions* as part of the CAI (1998a) model, in which relevant hypotheses are defined at the offence, activity and source levels. While the scientist is able to make inferences on the source and activity level, the ultimate question of guilt at the offence level lies with a judge or jury.

To make inferences and guide decision-making, logical reasoning processes such as multivariate analytical methods can be used (Dawid and Evett 1997). One of these methods is the use of Bayesian networks (BNs) (Fenton and Neil 2012; Taroni et al. 2014), which has been applied to the interpretation of various kinds of evidence, including DNA (Evett et al. 2002), fire incidents (Biedermann et al. 2005), gunshot residue (Biedermann et al. 2009; Fenton et al. 2014), glass (Zadora 2009), and document examinations (Biedermann et al. 2011). In BNs, one can graphically formalise the probabilistic (uncertain) relations between the hypotheses in question and the available evidence. These are represented as nodes, and the relations are formalised in node probability tables (NPTs). New evidence is incorporated by using Bayes’ theorem to update the previous (prior) belief in a hypothesis to a new (posterior) probability in the light of the strength of this evidence. Equivalently, it is also possible to compute the overall likelihood ratio (LR) of pieces of evidence (either individually or in combination) with respect to any particular hypothesis; the benefit of the LR approach is that it avoids the need to define prior probabilities for hypotheses that are not themselves conditioned on other hypotheses or evidence.

Despite the research successes, the Bayesian approach in a legal setting suffered a setback as a result of the case of R v. T (2010), which questioned the use of probabilistic approaches in court with regards to shoeprint evidence. This led to significant debates about the role of Bayesian approaches in presenting evidence types that do not have an established statistical base akin to DNA and more generally, how forensic evidence should be presented in court (Berger et al. 2011; Hamer 2012; Morrison 2012; Redmayne et al. 2011; Robertson et al. 2011). Thompson (2012) concluded that, in order to avoid such problems in the future, the forensic scientist should only receive necessary and specific information, valid scientific bases should accompany their presented evidence, their reasoning processes should be transparent and lastly, the results should be presented to legal professionals in an understandable way. This could all be better regulated by the use of LRs (Thompson 2012), although [as was demonstrated in (Fenton et al. 2014)], there are several important limitations and caveats that need to be considered when using the likelihood ratio to summarise the impact of evidence in a Bayesian argument. Ultimately, if strategic and cultural challenges (such as support for and understanding by the criminal justice system) can be overcome, the Bayesian approach could become the central method for the evaluation of evidence (Fenton et al. 2013a).

### Objectives

This paper aims to contribute to overcoming the aforementioned challenges by showing how BNs can be used to structure and represent the set of information and questions in the context of a criminal case, as a structural and transparent tool to evaluate the value of new evidence that is acquired in the stages prior to an appeal hearing. It presents a method to develop the BN that captures the relation between the acquired forensic evidence and the proposed hypotheses. The BN is subsequently used to help define the experimental studies necessary to evaluate the new evidence, to infer the evidentiary value of the newly acquired evidence, and to evaluate the evidence that is of most value to the hypothesis of interest.

### Case study

In the case we consider here, the defendant was convicted of attempted murder. In summary (the full details are provided in “Requirements and case pre-assessment” section), the young child of the defendant was taken to hospital with serious injuries after an alleged incident took place while the baby and the defendant were alone in a room. The defendant allegedly hurt the baby, resulting in blood stains belonging to the baby being found on the wall in the room. One of the key items of evidence presented by the prosecution was an audio recording of an emergency services call that the defendant made immediately after the incident. On the recording there were distinct sounds that the prosecution claimed to be linked to the criminal act, namely the defendant scraping blood off a wall. During the trial, the jury was asked to decide whether or not they could be sure of either of the alternative counts on the indictment: attempted murder or causing grievous bodily harm with intent. After the defendant was convicted of murder, questions were raised by the defence team that led to an appeal process being set in place, where the audio evidence was re-analysed by an audio expert. Following this, the most important sections in the grounds of appeal were:

- the prosecution theory concerning the recorded sound was presented during the trial as if it was an established fact without any evidence from an expert, and
- new reports of the defence expert undermine the contention that the sound in question must have been made by scraping blood off the wall.

### Content

This paper firstly provides an overview of the methods that are used to define the relevant propositions, create the network and evaluate the evidence in this case (“Methods” section). The subsequent sections present the requirements and case pre-assessment applied to the case study (“Requirements and case pre-assessment” section), the BN created using this information (“The BN models for the case” section), and the assessment of the evidentiary value of the new evidence (“Evaluation of the evidential values” section).

## Methods

### The propositions and the Bayesian network (BN)

All the original case materials were made available and were examined to identify the propositions, at different levels, that were of importance in the trial and subsequently addressed in the preparation for the appeal. Once the relevant propositions and required outcomes were set, a BN was created using the basic connective structures and building methods previously discussed in the literature (Fenton et al. 2013b; Taroni et al. 2004), where the presented propositions represent the arguments raised by the defence and prosecution in the original case. The BN was then extended by incorporating the newly gained information in preparation for the appeal and created using the freely available software package AgenaRisk (AgenaRisk 2015).

### Conditional probabilities and inference methods

When revising the probabilities for a pair of prosecution (*H*_*p*_) and defence (*H*_*d*_) hypotheses in the light of *n* items of evidence, the posterior probabilities $P (H_{p} |E1, \ldots , En)$ and $P (H_{d} |E1, \ldots , En)$ can be found using Bayes’ theorem, which can be derived from the general laws of conditional probability: the prior probability is multiplied with the support that the new evidence ($E1, \ldots , En$) provides for the hypotheses:

$$P\left( {H_{p} |E1, \ldots , En} \right) = \frac{{P(E1, \ldots , En|H_{p} )}}{P(E1, \ldots ,En)} \cdot P(H_{p} )$$

$$P\left( {H_{d} |E1, \ldots , En} \right) = \frac{{P(E1, \ldots , En|H_{d} )}}{P(E1, \ldots ,En)} \cdot P(H_{d} )$$

By dividing these probabilities, we get the ‘odds’ form of Bayes theorem which shows that the posterior odds are the product of the prior odds and the likelihood ratio.

$$\frac{{P\left( {H_{p} |E1, \ldots , En} \right)}}{{P\left( {H_{d} |E1, \ldots , En} \right)}} = \frac{{P(E1, \ldots , En|H_{p} )}}{{P(E1, \ldots , En|H_{d} )}} \cdot \frac{{P\left( {H_{p} } \right)}}{{P\left( {H_{d} } \right)}} = {\text{likelihood ratio }} \cdot {\text{prior odds}}$$

Providing that the pair of prosecution and defence hypotheses are mutually exclusive and exhaustive (meaning that *H*_*d*_ is equivalent to “not *H*_*p*_”) the likelihood ratio *on its own is a valid* measure of probative value of the evidence ($E1, \ldots , En$) on the pair of hypotheses (Fenton et al. 2014). Specifically, if the LR > 1 the evidence supports *H*_*p*_ (with higher values offering greater support) and if the LR < 1, the evidence supports *H*_*d*_ (with lower values offering greater support). This is very attractive for forensic scientists since it means they can assess the probative value of the evidence without having to provide any prior probability for hypothesis *H*_*p*_. However, while computing the LR is simple for a single piece of evidence [the expert only has to provide two likelihoods $P(E|H_{p} )$ and $P(E|H_{d})$], when there are multiple pieces of (possibly) dependent evidence along with other unknown but related hypotheses, the computation of the probabilities $P(E1, \ldots , En|H_{p} )$ and $P(E1, \ldots , En|H_{d}$) needed for the LR requires a full inference over the entire BN model that captures all of these dependencies.

In the BN model, we only have to provide ‘local’ likelihoods of individual evidence nodes given the parents—these can be extracted from the case information where available, and stored in the NPTs. Once the BN structure is created (showing the conditional dependent relations) and these ‘local’ likelihood values are provided for the NPTs, the over-all likelihood ratio for any set of evidence with respect to any hypothesis node can be obtained by running the model in BN software (e.g., AgenaRisk, Hugin). Specifically for evidence $E = \left\{ {E 1, \ldots ,En} \right\}$ when we run the model with observations for that evidence, the model computes (for any hypothesis node $H$), the value $P\left( {H|E} \right)$ [and $P({\text{not}} H|E)$, which must be $1 - P\left( {H|E} \right)$]. But, rearranging the formula for the odds version of Bayes, we get:

$$LR = \frac{P(E|H)}{{P(E|{\text{not}} H)}} = \frac{P(H|E)}{{P({\text{not}} H|E)}} \cdot \frac{{P({\text{not}} H)}}{P(H)}$$

where $P\left( H \right)$ is the marginal prior probability value for H when the model is running without any observation for the evidence nodes. So, the BN computation provides the correct LR value for E with respect to H.

### Representation of case information

To translate the verbal scales used in the case documentation to numerical probabilities, the translation table presented by Morgan (2009) was used. Even though it is recognized that this translation is still subjective, it is merely used to show how BNs can be used in cases where likelihoods are or can actually be reliably assigned. However, if there is insufficient information regarding such probabilities (such as a lack of databases or experimental studies), the probabilities are represented as a variable which can be studied across a range of possible values.

### Sensitivity analysis

In order to investigate the evidential value of each item of evidence to the hypothesis of interest a sensitivity analysis was performed. Such an analysis allows one to identify those with the strongest evidential value, by calculating and comparing the probabilities of the target node with and without assumed states for (a combination of) the evidence nodes. For example, by comparing $P\left( {H_{p} } \right)$, $P(H_{p} |E 1 = e 1)$, and $P(H_{p} |E 2 = e 2)$. The results of such changes will be visualised in a so-called *tornado graph*.

Additionally, the effect when the missing conditional probabilities are actually obtained was assessed. This method was designed to result in a transparent reasoning process which is understandable by legal professionals and jurors, and addresses some of the issues raised by Thompson (2012) which are summarised in “Approaches to interpretation” section.

## Requirements and case pre-assessment

### Case information

From the case documentation, it follows that, in relation to the audio evidence, the following information can be observed:

- A baby was injured during an incident on the top floor of a house
- Blood from the baby was found on the wall in one of the rooms upstairs
- On an audio recording of the emergency telephone call made by the suspect, a scraping sound (allegedly indicating scraping blood off a wall) can be heard (hereafter referred to as sound 1 at time $t_{ 1}$)
- The suspect was charged with attempted murder.

The audio evidence played a significant role in the trial. However, the assessment of observing the evidence that would be expected if the suspect did indeed scrape blood off the wall was not supported by any empirical studies or past experiences. During the appeal preparation process, the recorded emergency call was re-analysed by an audio expert on behalf of the defence, and four other sounds were identified on the same recording that, according to the expert, *showed similarities* to sound 1. In particular, one of these sounds (referred to as *sound 2*) was of interest because of background noise that could be heard simultaneously at this time $t_{2}$. This expert statement provides evidence for both the activity causing sound 1 and the activity causing sound 2. The background noise was presumed to be the television, which was located in a different room to where the prosecution argued the scraping of the blood took place. In summary, the following additional information was added in the preparation for the appeal:

- The proposition that the suspect was rubbing his face was given as an alternative explanation by the defence for the explanation of sound 1 (in what follows we assume that these two hypotheses are mutually exclusive)
- A second sound (sound 2) was noted on the emergency recording, which, according to the audio expert, *showed similarities* to sound 1
- During this second sound, the TV (located downstairs) could be heard simultaneously on the emergency recording
- A statement by the police reads that the suspect was frequently rubbing his face in their presence

### Questions

Following the available information, the questions of interest (from an offence to a source level) to the case can be defined as:

- Did the suspect attempt to murder the victim?
- Did the suspect inflict any injuries on the victim?
- Did the suspect scrape blood off the wall during the emergency call causing sound 1 (time $t_{ 1}$)?
- Are sound 1 and 2 similar?
- Can the TV be heard during sound 2 (time $t_{ 2}$) if the suspect was positioned in the room upstairs?

Clearly, the formulation of the questions in the case assessment stage will impact the structure of the BN. For example, asking (as was the case in the trial)

“were the sounds *similar*”

is different from asking:

“were the sounds produced by the same activity?”

unless a similarity is defined as such. This highlights the importance of precisely defining the meaning of the terms used in the assessment.

### Propositions

The ultimate question is whether the suspect attempted to murder the victim—as was the final statement in the trial. The other counts that were presented to the jury were innocence or causing GBH. Factors of relevance (and discriminatory power) to the three counts are the degree of preparation, motive, opportunity, intent, and whether or not the criminal action actually took place. It is argued by Fenton et al. (2013b) that because opportunity, motive and intent are causes or pre-conditions for the offender to commit the crime, they should be parental nodes in a BN model. The same can be said about the degree of preparation (or malicious aforethought), which has a significant role in distinguishing between the counts of attempted murder and causing GBH. However, keeping in mind the objective of the appeal—to provide evidence which might influence the initial decision—only the propositions that are of relevance to the new evidence needs to be reassessed and is of relevance to the defence. The main forensic evidence available in this case relates to the question of whether the suspect scraped blood off the wall (which is subsequently said to be linked to the act of inflicting injuries on the victim). Hence, our BN model focuses only on source level hypotheses, ignoring the offence level hypotheses and the associated difficulties of modelling factors like opportunity, motive and intent.

The evidence presented in the trial to support the scraping/rubbing propositions was that of the sound, which is *consistent with the suspect scraping blood*. Ideally, of course, these hypotheses should be accompanied by an analysis that discusses the validity and accuracy of the evidence presented. However, as this was not done in the trial, it will therefore not be taken into consideration in this model. Statements about the presence of the other evidence types are defined similarly. Thus, the propositions presented in this case are:

- *The suspect scraped blood off the wall at time* $t_{ 1}$, and the alternative *the suspect rubbed his face at time* $t_{1}$.
- *A sound that is consistent with the suspect scraping blood at time* $t_{ 1}$ *is observed on the recording,* and *a sound that is consistent with the suspect scraping blood at time* $t_{ 1}$ *is not observed on the recording,*
- The suspect scraped blood off the wall at time $t_{2}$, and *the suspect rubbed his face at time* $t_{ 2}$.
- *A sound that is consistent with the suspect scraping blood at time* $t_{ 2}$ *is observed on the recording, and a sound that is consistent with the suspect scraping blood at time* $t_{ 2}$ *is not observed on the recording,*
- *Sound 1 and sound 2 are similar,* and *sound 1* and *sound 2 are not similar.*
- The TV can be observed on the recording at time $t_{2}$, and *the TV cannot be observed on the recording at time* $t_{ 2}$,
- *The police observed a frequent rubbing activity by the suspect,* and *the police did not observe a frequent rubbing activity by the suspect.*

These propositions highlight some important considerations in the inference process when one wants to assess LRs applied to real cases. The prosecution (scraping activity) and defence (rubbing activity) hypotheses are assumed to be mutually exclusive but not exhaustive, since the proposition that it is caused by another activity is not considered (Fenton et al. 2014). In theory, such a third hypotheses of *‘other activity’* could have been included in the activity nodes, if it were not for the fact that questions such as ‘*what is the probability of observing a similarity between a sound caused by scraping and one caused by any other activity*’ are not possible to address using empirical data. Because of this, for example, a LR of 1 does not indicate that the evidence does not have any probative value: even though it might reduce the posterior probabilities of the hypotheses by equal amounts, it could increase the posterior probability of the third ‘other’ hypothesis (Fenton et al. 2014). Therefore, the LR will merely be used to determine whether the evidence provides more or less (or equal) support for one of the hypotheses over the other, rather than that it represents the aforementioned *probative* value of the evidence. This emphasizes the importance of formulating and thinking about the case information and desired and feasible outcomes before defining questions and hypotheses by using, for example, the Case Assessment model.

## The BN models for the case

The relationship between the propositions defined in “Propositions” section and the evidence that can support or refute these hypotheses are now made explicit in the BN models.

### The trial

**Fig. 1:**
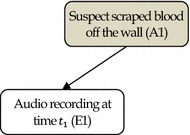
Bayesian network capturing the trial arguments related to the audio evidence

In the trial, the prosecution hypothesis that the suspect scraped blood off the wall causing a sound at time $t_{ 1}$ $\left( {a 1_{p} } \right)$ was used to support the proposition that the suspect might have inflicted injuries to the victim. At this stage, the defence did not have an alternative state other than that ‘the suspect did not scrape blood off the wall at time $t_{ 1}$' $\left( {a 1_{d} } \right)$. To support their hypothesis, the prosecution presented an audio recording (E1) on which sound 1 could allegedly be heard. The dependency between these is causal in such a way that the scraping activity causes the sound to be created, and therefore recordable and observable. This item of evidence can *be* heard $\left( {e 1} \right)$, or simply *not be* heard $(\bar{e}1)$ to support or oppose the scraping proposition, and does not depend on the standpoint of either of the parties. The network that captures these nodes and the relation between them can be found in Fig. 1.

### The appeal

**Fig. 2:**
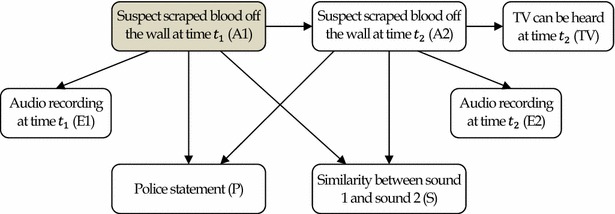
Bayesian network capturing the hypotheses and acquired evidence in the case

In the preparation for the appeal, the state for the defence proposition $a 1_{d}$ changed when they provided the alternative explanation that the suspect was rubbing his face at $t_{ 1}$. The second sound $\left( {A 2} \right)$ can now be added to the network with the accompanying evidence $\left( {E 2} \right)$. The assumption is made that the activities causing both sounds are dependent in such a way that if the suspect is acting in a certain way at time $t_{ 1}$, it is reasonable to assume this is related to the activity at time $t_{2}$ ^.^ Following the case information, there is a link between whether or not the television could be heard $\left( {TV} \right)$ and the activity causing the second sound, due to the distance between the location of the TV and the specific wall. Additionally, the evidence on the similarity $\left( S \right)$ between the sounds is conditionally dependent on, logically, the activities that caused both sound 1 $\left( {A 1} \right)$ and sound 2 $\left( {A 2} \right)$. The last piece of evidence relevant to these propositions is a statement given by the police $\left( P \right)$ that a police officer *observed a frequent rubbing activity by the suspect*. The new network is shown in Fig. 2, highlighting the node $\left( {A 1} \right)$ that is of ultimate relevance to the appeal.

### The conditional probabilities

In order to perform the necessary Bayesian inference to determine the impact of the evidence we must provide all of the NPTs for the model. Typically, these are provided either from data, experimentation or expert judgment. In the absence of substantive data, in what follows we consider ranges of values where relevant and perform sensitivity analysis over them, relying on the wording used in the case documentation and the verbal scale translation table shown in Table 1. As discussed before, these probabilities are merely used to how BNs can be used in cases where probabilities can be assigned based on more robust empirical grounds.

**Table 1:** Probability ranges for verbal descriptions, taken from Morgan (2009)

Verbal term	Probability range
Virtually certain	>0.99
Very likely	0.9–0.99
Likely	0.66–0.9
Medium likelihood	0.33–0.66
Unlikely	0.1–0.33
Very unlikely	0.01–0.1
Exceptionally unlikely	<0.01

#### The activity nodes A1 and A2

The prior probability of activity 1 being scraping is unknown, since this may depend on background information that is not available in this case. Additionally, it was assumed that there is a dependency between the two activities where it is a bit more likely that A2 (with states $a 2_{p}$ and $a 2_{d}$) has a certain state given that this was also the case during the first activity:

$$P(A1 = a1_{p} ) = x$$

$$P(A2 = a2_{p} |A1 = a1_{p} ) = P(A2 = a2_{d} |A1 = a1_{d} ) = 0.6$$

Note that in the remainder of this paper only the (unique) state names will be used to refer to the state of the nodes in the equations to maintain conciseness [e.g. $P(A 2 = a 2_{p} |A 1 = a 1_{p} ) = P\left( {a 2_{p} |a 1_{p} } \right)$].

#### The audio evidence E1 and E2

The first evidence to assess are the sounds that are audible on the recording, which will be modelled in the same way and are claimed to be scraping sounds by the prosecution. Therefore, if the suspect was scraping the wall at the particular times (node states $a 1_{p}$ and $a 2_{p}$), it is expected that the audible sound appears to be scraping: $E 1$ and $E 2$ are *very likely* to be present (node states $e 1$ and $e 2$):

$$P(e1| a1_{p} ) = P(e2| a2_{p} ) = 0.95$$

If, however, the suspect was rubbing his face at the particular times ($a 1_{d}$ and $a 2_{d}$), not much can be said about observing a scraping sound since the required information for the conditional probability is unavailable:

$$P(e1| a1_{d} ) = P(e2| a2_{d} ) = y$$

#### Evidence of similarity S

Following the expert statement of the audio expert, a certain level of *similarity* is observed between sound 1 and 2 (with states $S = s$ and $S = \bar{s}$). The probability of observing a similarity ($S = s$) between these sounds if they are produced by the same activity is assumed to be *almost certain*:

$$P(s| a1_{d} , a2_{d} ) = P(s| a1_{p} , a2_{p} ) = 0.99$$

The probability of observing a similarity between the two sounds given that each of the sounds are caused by a *different* activity is not assessed. This information is however crucial for the likelihood ratio of the ultimate hypothesis in this case (whether the suspect scraped blood off the wall) and therefore assessing the support of evidence of the expert witness statement on the degree of similarity:

$$P(s| a1_{d} , a2_{p} ) = P(s| a1_{p} , a2_{d} ) = z$$

In order to obtain these probabilities, experimental studies should have been performed in which the case situation is simulated and the different sounds resulting compared.

#### Police statement P

The probability of the police observing a rubbing activity and writing this into a statement (states $P = p$ and $P = \bar{p}$) is higher if at both times the suspect rubbed his face than if he only did this at one point (e.g. an increase by 0.05), and less likely if the suspect was scratching the wall both times (e.g. a decrease by 0.05). Additionally, knowing that the suspect was scraping the wall at both points in time does not give an indication as to the likelihood of observing P. Since the relative values in the NPT are more important rather than the exact value of the conditional probability, the following assumptions are made:

$$P(p|a1_{p} , a2_{p} ) < P(p| a1_{d} , a2_{p} ) = P(p|a1_{p} , a2_{d} ) < P(p| a1_{d} , a2_{d} )$$

$$P(p| a1_{p} , a2_{p} ) \; = \; 0.5$$

#### Evidence regarding the background noise from the TV

Because the television was, according to the case records, positioned in a downstairs room relatively far away from the upstairs bedroom in which the scraping allegedly took place, it is considered to be *a lot more likely* that the television could not be heard $(TV = \overline{tv} )$ than that it could (*TV* = *tv*), given that the suspect was scraping the wall at time *t*2:

$$P(tv| a2_{p} ) = 0.1$$

$$P(\overline{tv} | a2_{p} ) = 0.9$$

**Fig. 3:**
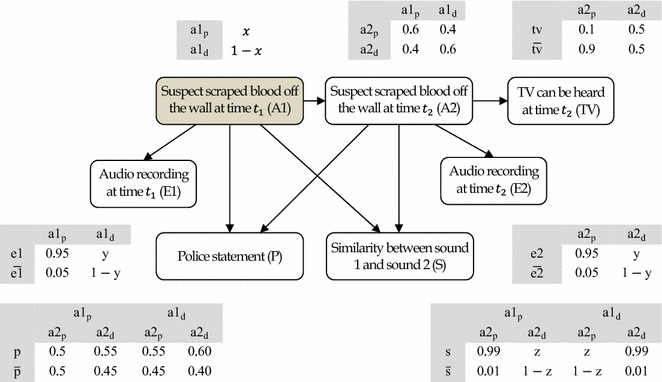
Bayesian network for the appeal case including the conditional probabilities

The full set of (conditional) probabilities for the BN model is presented in Fig. 3.

## Evaluation of the evidential values

It follows from the BN in Fig. 3 that the probability of *A*1 is conditional and depends on different factors that need to be assessed. Even though there is scope for disagreement regarding the probabilities and assumptions, it is possible to make inferences from the BN that can aid the interpretations of evidence within the case.

### Evidential value and experimental studies

**Fig. 4:**
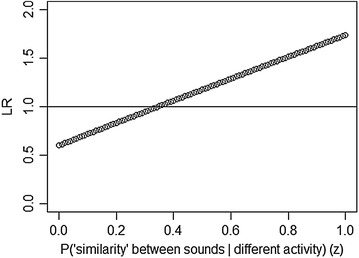
LR of the prosecution (scraping) and defence (rubbing) hypotheses for values of z

The question that is of interest in a legal setting is: ‘*does the new appeal evidence change the belief in* $a1_{P}$’? And if so, given the aim of the defence for the appeal: ‘*does the presence of the new appeal evidence lower the probability of* $a1_{P}$’? The variables $x, y$ and *z* discussed in the previous section are different in such a way that *x* (the prior probability of $a1_{P}$) is based on human considerations while attempts can be made to approach a more precise value of *y* and *z* through experimental studies. However, because the expert statement regarding the audio evidence does not provide any information about the likelihood of observing a similarity between two sounds that are caused by a different activity (*z*), key evidence is missing to come to any conclusion. Figure 4 shows how, for *y* = 0.5, a difference in the *z*-value changes the LR to such an extent that the evidence supports different hypotheses depending on this value:

- For 0 ≤ *z* < 0.35, *LR* < 1, indicating that the evidence supports the hypothesis that the suspect was undertaking a normal behaviour, which is beneficial for the defence.
- For 0.35 < *z* < 1.0, *LR* > 1, indicating that the evidence supports the hypothesis that the suspect was undertaking a ‘suspicious’ behaviour, which is beneficial for the prosecution.

**Fig. 5:**
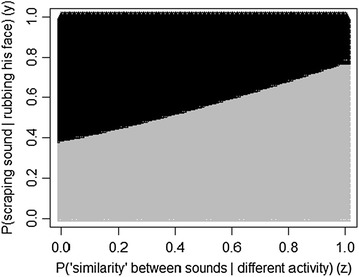
The evidence either supports the defence (LR < 1, *dark points*) or the prosecution (LR > 1, *light points*) depending on the values for the unknowns $\varvec{y}$ and $\varvec{z}$

Following this, Fig. 5 shows that the evidence either supports the defence (*LR* < 1, dark points) or the prosecution (*LR* > 1, light points) depending on the values for the unknowns *y* and *z*. This is especially the case for higher values of *y*.

**Fig. 6:**
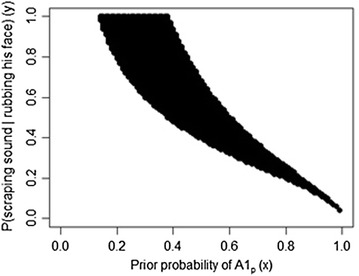
Graph showing the cases where the evidence can benefit both the defence and prosecution hypotheses. A *point* in the graph indicates that, for that specific combination of the x and y value, a variation in the z-value can change the beneficiary of the evidence

As noted before, the LR only provides information about to what extent the evidence supports one of the hypotheses over the other. However, the court is interested in addressing which of the two hypotheses is more likely based on the evidence (confusing these two is labelled the *prosecutors fallacy*). In order to do this, we need to incorporate the prior probabilities of the prosecutor and defence hypotheses, a value which can vary depending on the background information that one assumes to be present $(P(H_{p} = x))$. Figure 6 shows the results of varying the prior assumption *x* and the (currently) unknown value of *y*. A point in the graph indicates that for that specific combination of *x* and *y*, varying the value of the other unknown, *z*, can cause a switch in which sides’ hypothesis is most likely based on the evidence presented at the trial. This is especially the case for lower *x* values (which would favour the defence) and higher *y* values (also in favour of the defence). Because of this, the defence should be very careful in presenting the new evidence in its favour when insufficient information is given by the expert with regards to the other unknown variables. However, if experimental studies were to show that the two possible activities do produce dissimilar sounds (and the value of *z* tends towards zero), the expert evidence reduces the posterior probability of the defendant scraping the wall greatly, which was the aim of the defence. Experimental studies are therefore needed to offer the empirical evidence base to establish the missing parameters in a Bayesian network, leaving less room for speculations.

### Most influential items of evidence

A sensitivity analysis was performed to identify the items of evidence that have the most impact on the hypothesis A1 (whether the activity that caused the first sound was related or unrelated to the crime). Because *y* cannot be determined using the current case information, it is assumed that, given that the activity was rubbing, no distinction can be made between the two possible states of the evidence node (a *y*-value of 0.5). The prior probability *x* of (*A*1 = *a*1_*p*_) is also set to 0.5 since no background evidence is assumed to be present before the evidence was presented at the trial. Since the defence assumed a low value for *z*, a value of 0.1 is used. Note that these values are only selected in order to show the effect and interaction of the items of evidence and are not used to show the evidential value.

**Fig. 7:**
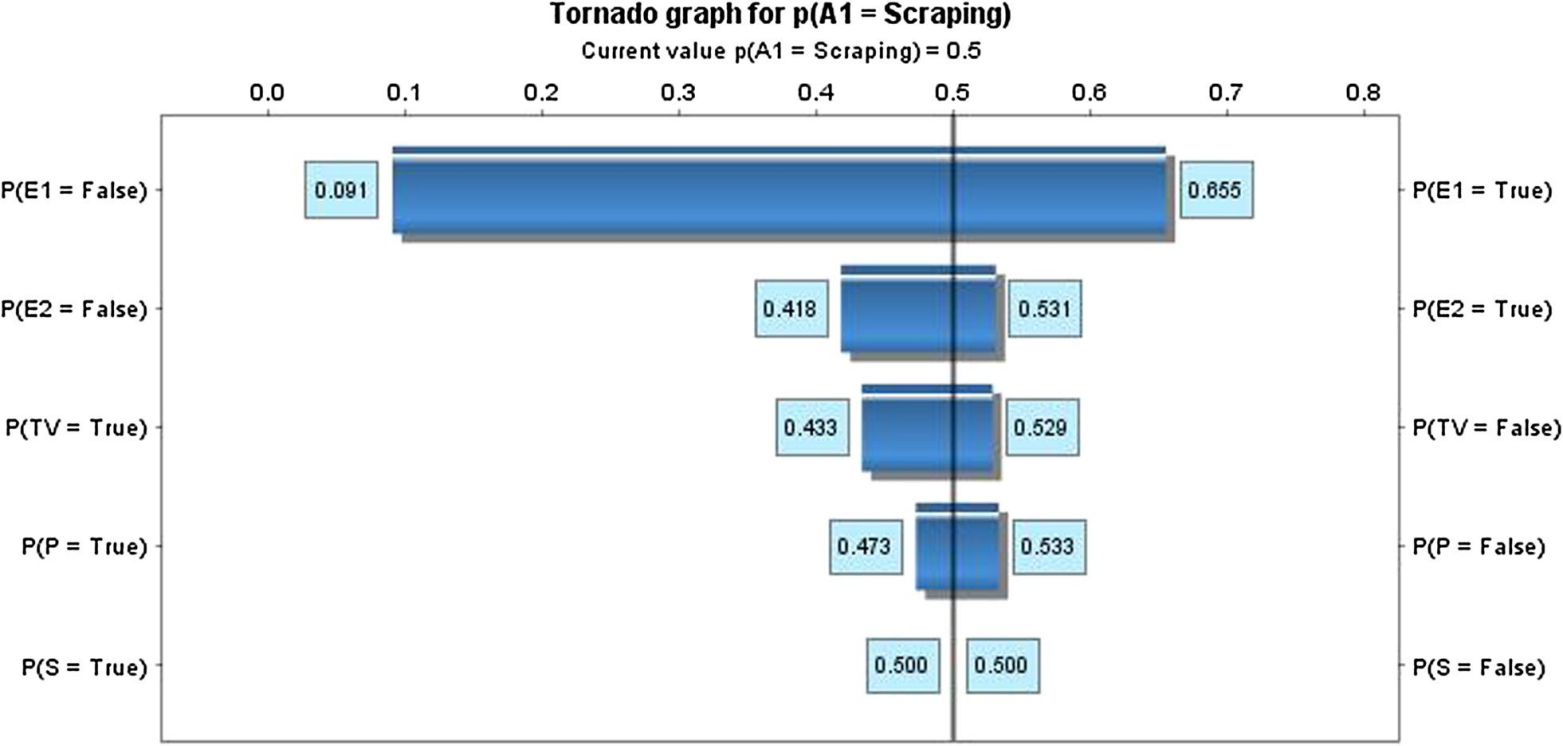
Results of the sensitivity analysis showing which nodes have the most influence in changing the belief in the prosecution hypothesis of A1

The results of the sensitivity analysis are presented in the form of a tornado graph, see Fig. 7. The vertical line shows the prior probability where no evidence is assumed to be present yet (*P*(*a*1_*p*_) = 0.5). For each of the other nodes in the network, the bars represent the extent an assumption about their state impacts the belief that the suspect was scraping blood off the wall at time *t*_1_. It follows from Fig. 7 that the probability of the suspect scraping the wall is most impacted by the presence of the two sounds, followed by the observations regarding the TV and the police statement. In this case, the evidence of similarity has negligible impact, since no assumptions are made regarding the presence of the two sounds.

**Fig. 8:**
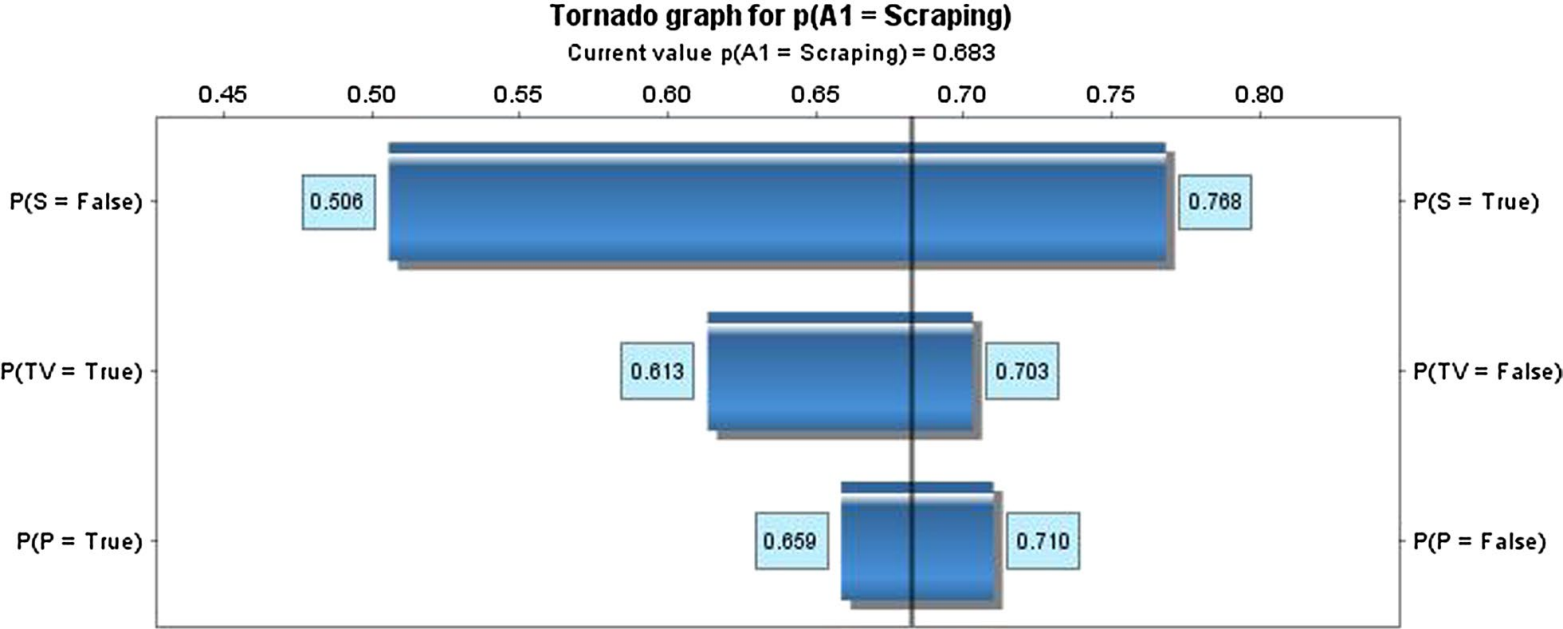
Results of the sensitivity analysis showing the variation of *a1*_*p*_ for fixed states of *E*1 and *E*2

**Fig. 9:**
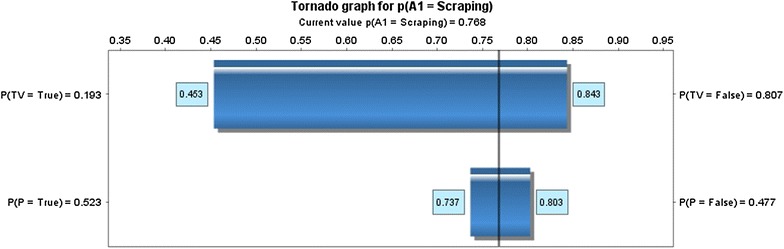
Results of the sensitivity analysis showing the variation of *a1*_*p*_ for fixed states of *E*1*, E*2 *and S*

The subsequent assumption is made that the items of evidence E1 and E2 are observed to draw further inferences. Figure 8 shows the results of the sensitivity analysis. Because of the chosen state of *E*1 and *E*2, the evidence now favours the prosecution without considering any of the other evidence: $P(a1_{p} | e1,e2) = 0.68$. It follows that the evidence of similarity between the sounds is most important, which can vary the probability of *a*1_*p*_ between 0.51 and 0.77. Subsequently, the evidence related to the television and the observation of the police statement are less significant in changing the belief in *a*1_*p*_. However, because the value of *a*2_*p*_ is not known, the related items of evidence are dependent where a change in the belief of S influences the state of the television evidence. To further assess this issue, a similar analysis is performed where the evidence of similarity is assumed to be present (Fig. 9). The posterior probability of $a1_{p}$ increased to 0.77 without considering the states of the police statement and TV evidence. It follows that the television evidence can change the probability of *a*1_*p*_ to lower (0.45) or higher (0.84) than its prior (0.5). This sensitivity analysis thus shows that the inclusion or exclusion of items of evidence in the interpretation process can have a great impact on the significance of other items. Without the presence of the television evidence, the evidence on the similarities brought forward by the defence actually increases the probability of the prosecution hypothesis.

## Conclusion

This study has demonstrated how the Bayesian framework of reasoning can be used to explore how items of evidence are related to each other and to the relevant hypotheses, illustrated by a real case. It highlighted the importance of assessing the information and defining the questions and propositions at different levels before creating the BN model. When one makes reasonable and justifiable decisions regarding aspects such as conditional independence, integrity of expert evidence and assumptions that are made in favour of the defence or prosecution, the BN can be a useful tool to provide transparent and workable results regarding the relevance of new items of evidence.

The changes in the key posterior probability was shown to be dependent on the different assumptions, ultimately showing that the incomplete defence expert evidence could also work to weaken the defence case. More specifically, the evidence about similarity of the sounds is the most valuable when aiming to address the activity level proposition, followed by the observation of the TV evidence. Because of this, it is important that experimental studies are undertaken in order to define the probabilistic relationships (Morgan et al. 2009), a process which can be guided using the presented methods. Additionally, it was shown that the importance of certain items of evidence is influenced by the true state of others, illustrating the importance of evaluating the dependencies and incorporating all the relevant available evidence.

This research therefore addresses a critical issue within the forensic sciences. The importance of presenting—in a holistic, transparent and reproducible manner—all the relevant evidence and hypotheses is, we believe, paramount for optimising the use of valuable forensic evidence in court. This approach enables judges and juries to evaluate the weight of not only individual items of evidence in a case but also identify the interaction of different items of evidence within that case to be able to draw conclusions. Such an approach offers a significant step that enables forensic science to address a number of the critiques that have been made in recent years, and to ensure that the value of physical evidence continues to be realised in forensic investigations and the pursuit of justice.

## Abbreviations

BN: Bayesian network
CAI: case assessment and interpretation
NPT: node probability table
LR: likelihood ratio
